# Evolutionary aspects of non-metastatic breast cancer after primary treatment in a sub-Saharan African setting: a 16-year retrospective review at the Douala general hospital, Cameroon

**DOI:** 10.1186/s12885-017-3984-z

**Published:** 2018-01-05

**Authors:** Bibiana Ateh Nzeangung, Martin Essomba Biwole, Benjamin Momo Kadia, Ndemazie Nkafu Bechem, Christian Akem Dimala, Albert Mouelle Sone

**Affiliations:** 1 Faculty of Medicine and Pharmaceutical Sciences, University of Douala, Douala, Cameroon; 2 Foumbot District Hospital, Foumbot, Cameroon; 3 Grace Community Health and Development Association, Kumba, Cameroon; 4 Faculty of Health Sciences, University of Buea, Buea, Cameroon; 5 School of Health Sciences, University of Nottingham, Nottingham, UK; 6 Health and Human Development (2HD) Research Network, Douala, Cameroon; 7 Faculty of Epidemiology and Population Health, London School of Hygiene and Tropical Medicine, London, UK; 8 Department of Orthopaedics, Southend University Hospital, Essex, UK

**Keywords:** Evolution, non-metastatic, breast cancer, sub-Saharan Africa

## Abstract

**Background:**

Breast cancer has a high case fatality rate in sub-Saharan Africa, and this is chiefly because of late detection and inadequate treatment resources. Progressive renovations in diagnostic and management modalities of non-metastatic breast cancer (NMBC) have been noted in the region but there is paucity of data describing the clinical progress of patients with NMBC. This study sought to determine the rates of local relapse, distant metastasis and sequelae and the time span from initial treatment to the occurrence of these adverse events among patients with NMBC.

**Methods:**

This was a retrospective review of medical records of patients with histologically confirmed NMBC at the department of radiation therapy and oncology of the Douala General Hospital in Cameroon from the January 1997 to December 2012 period. Clinicopathological and treatment characteristics as well as occurrences of adverse events were studied.

**Results:**

A total of 260 cases were reviewed of which 224/260 (86.2%) had invasive ductal carcinoma. Surgery was performed on 258/260 cases (99.2%) with 187/258 (72.5%) being modified radical mastectomies. Various treatment combinations were used in up to 228/260 patients (87.5%) while surgery alone was the treatment in the remaining 32 cases (12.5%). Metastasis occurred in 142/260 cases (54.6%) of which 68/142 (26.2%) were local relapses and 74/142 (28.5%) were distant metastases. Among the cases of distant metastasis, 9.2% were bone, 8.5% lungs, 6.9% nodal, and 5.4% brain. Metastasis to multiple organs was noted in 4.7% of these cases. The median periods of occurrence of local relapse and distant metastases were 13 and 12 months respectively. Sequelae occurred in 26/260 cases (10%) and were noted after an average of 30 months. The main sequelae were lymphoedema (6.5%) and lung fibrosis (1.5%). At the end of the period under review, 118/260 patients (45.4%) were alive and disease-free with a median follow up time of 24 months.

**Conclusions:**

Adverse events were frequent among patients who received primary treatment for NMBC. Available cancer therapeutic modalities ought to be supplemented with efficient strategies of follow-up and monitoring so as to optimize the care provided to these patients and improve on their survival.

## Background

Breast cancer is the leading cancer among the female population in the majority of African countries [1]. More than half of women diagnosed with breast cancer in Africa die of the disease [2]. The incidence of breast cancer in Africa continues to increase and is projected to double by 2050 [3]. The rise in the rate of breast cancer has been associated with the increased prevalence of risk factors such as early menarche, late child bearing, and obesity, as well as an increase in breast cancer detection due to gradual health infrastructural development [4]. In Cameroon, the 2012 Yaounde cancer registry revealed that breast cancer accounted for about 30% of all cancers diagnosed in the community [5].

The case fatality rate of breast cancer in low and middle-income settings of sub-Saharan Africa appears to considerably exceed that in high-income countries and this is mainly as a result of advanced disease at the time of diagnosis and inadequate management resources that prevail in sub-Saharan Africa [6]. Recently, progressive refinements in breast cancer detection and treatment strategies paralleled by improvements in non-metastatic breast cancer (NMBC) management modalities have been observed in the region [7]. In Cameroon, few hospitals such as the Yaounde General Hospital and Douala General Hospital already posess multidisciplinary treatment plans [8]. Notwithstanding these advancements, NMBC patients who receive treatment are not spared of adverse events. These adverse events could be related to breast cancer itself, cancer therapy or both [9]. There is a major paucity of data regarding morbidity associated with such adverse events and it is therefore imperative to provide recent and reliable data characterizing the clinical progress of patients with NMBC after initial treatment in sub-Saharan Africa.

The objectives of this study were to determine the rates of local relapse, distant metastasis and sequelae and the time interval from initial treatment to the occurrence of these adverse events. The ultimate goal of the study is to provide data that will enable cancer treatment providers in sub-Saharan Africa to optimize the care given to patients with NMBC so as to improve on the survival of these patients.

## Methods

### Ethical considerations

This study was approved by the Institutional Ethics Committee for Human Health Research of the Faculty of Medicine and Pharmaceutical Sciences, University of Douala, Cameroon. Informed consent was waived by reason of the retrospective nature of the study.

### Study design, study period and setting

This was a descriptive retrospective study conducted at the radiotherapy and oncology department of the Douala General Hospital, which is a tertiary health care facility in the Littoral region in Cameroon. This hospital is one of the main tertiary healthcare facilities in the country that offers a complete treatment plan for breast cancer.

### Study population and sampling

The medical records of patients with histologically diagnosed non-metastatic breast cancer (including lymphoma) from January 1997 to December 2012 were reviewed. NMBC included early breast cancer (stage 0, I, II) and locally advanced breast cancer (Stage III) cases [9].

Inclusion criteria: Patients who had complete remission after primary treatment with curative intent. Complete remission was defined as absence of symptoms and signs of breast cancer, associated normalization of radiological and biochemical indices after treatment.

Exclusion criteria: Patients whose medical records did not meet up to 90% of the information required for the study.

### Study procedures and variables

The medical records of breast cancer patients treated at the radiation therapy service were retrieved and eligible cases were selected based on the predefined inclusion and exclusion criteria. A structured data collection form was used to record variables of interest. The clinicopathological data recorded per patient included demographic data: age and gender, historical data: menarche, menopausal status and parity, NMBC details: tumour size, and histological data. Treatment strategies for NMBC were categorized as surgery, radiotherapy, chemotherapy, hormone therapy, a combination of these or none. Adverse events, i.e., local relapse, distant metastasis, and sequelae after primary treatment were recorded. The follow-up period was considered as the period from diagnosis of breast cancer to the date when an adverse event was recorded. With regards to adverse events, metastatic recurrences were described as either local or distant. Local relapse (local recurrence) was defined as tumour recurrence in the ipsilateral lymph nodes (nodal recurrence), breast or chest wall while distant metastasis referred to tumour recurrence in sites remote from the breasts such as the lungs, bones, liver, and brain. Sequelae were defined as adverse events which are inherent to specific therapy, for example, lymphoedema secondary to surgical clearance of axillary lymph nodes.

### Statistical analysis

Statistical analysis was done with Epi Info version 7 statistical software. Descriptive statistics were depicted using absolute numbers, percentages, ranges and means where appropriate.

### Reporting

The study was reported using the STROBE guidelines.

## Results

The medical records of 568 patients diagnosed with breast cancer were reviewed amongst which 260 cases fulfilled the eligibility criteria, giving an inclusion rate of 45.8%.

### Clinicopathological characteristics

The ages of patients ranged from 21 to 90 years; with a mean age of 47.44 ± 11.8 years. Two hundred and fifty four patients (97.70%) were females while 6 patients (2.31%) were males. For the 254 women, the age at menarche ranged from 9 to 21 years with a mean of 13.4 ± 2.1 years. The majority of the women (87.5%) had normal menarche. Gravidity varied between 0 and 13 with a median of 5.0 for the 252 women whose records had the relevant information. Parity ranged from 0 to 11, with a mean of 4.2 ± 2.8. Breastfeeding was practiced in 88.2% of all the women, with the duration ranging from 3 to 18 months postpartum. Menopause was reported in 119/254 women (46.9%) at the time of diagnosis. The demographic and historical characteristics of the study population are summarized on Table 1.

**Table 1:** Demographic and historical characteristics of the study population

Variable	Category	Frequency (%)
Gender	Female	254(97.7)
	Male	6(2.31)
		***260(100)***
Age at diagnosis(in years)	21–30	15(5.8)
	31–40	59(22.7)
	41–50	100(38.5)
	51–60	47(18.1)
	>60	39(15.0)
		***260(100)***
Age at menarche (years)	<11	8(3.1)
	12–15	222(87.5)
	>15	24(9.4)
		***254(100)***
History of contraceptive pills	Yes	25(9.8)
	No	229(90.2)
		***254(100)***
Parity	≤1	56(22.0)
	2–5	115(45.3)
	>5	83(32.7)
		***254(100)***
Menopause at diagnosis	Yes	119(46.9)
	No	135(53.2)
		***254(100)***
Breastfeeding practice	Yes	224(88.2)
	No	30(11.8)
		***254(100)***
Family History of breast cancer	Yes	12(28.6)
	No	30(71.4)
		***42(100)***

The entries in boldface/italics represent the total case files for which information on the relevant variable was available

The left breast was affected in 152/260 (58.5%) of the cases compared with 107/260 (41.2%) for the right breast. Among the 256 cases with records on breast cancer location, the upper outer quadrant of the breast was involved in as many as 210 (82%), while only 6 (2.3%) had an affected lower inner quadrant. Relevant information on the topography of the lesions on the breast was available for 42 patients among whom 34/42 (81.0%) had unifocal lesions while multifocal lesions were noted in 8/42 (19.1%). Locally advanced (T3 and T4) breast cancer was noted in up to 170 (65.4%) of the 260 cases included. Invasive ductal carcinoma was the histological diagnosis in up to 224/260 (86.5%) of all cases. The clinicopathological characteristics of our cohort are summarized on Table 2.

**Table 2:** Breast cancer characteristics in study population

Variable	Range	Frequency (%)
Tumour location		
1. Affected quadrant		
Upper outer quadrant		210(82)
Upper inner quadrant		19(7.4)
Subareolar		14(5.5)
Lower outer quadrant		7(2.7)
Lower inner quadrant		6(2.3)
		***256(100)***
2. Affected breast		
Left		152(58.5)
Right		107(41.2)
Bilateral		1(0.4)
		***260(100)***
3. Area of lesion		
Unifocal		34(81.0)
Multifocal		8(19.1)
		***42(100)***
Tumour classification		
T0		4(1.5)
T1		26(10.1)
T2		60(23.2)
T3		98(37.7)
T4		72(27.5)
		***260(100)***
Histological type		
Invasive ductal carcinoma		224(86.2)
Invasive lobular carcinoma		12(4.6)
Intraductal carcinoma		8(3.1)
Invasive medullary carcinoma		6(2.3)
Apocrine carcinoma		3(1.2)
Comedo carcinoma		3(1.2)
Lymphoma		1(0.4)
Others		3(1.2)
		***260(100)***
Analysed lymph nodes ^a^	1–5	13(35.1)
	6–10	12(32.4)
	11–15	11(29.7)
	16–22	1(2.7)
		***37(100)***
Positive lymph nodes ^b^	0	5 (13.5)
	1–3	14 (37.8)
	≥4	18 (48.6)
		***37 (100)***

^a^Reported for patients with information on lymph node analysis in case file;

^b^Positive nodes among the 37 cases with information on lymph node analysis. Mean number of positive nodes was estimated at

The entries in boldface/italics represent the total case files for which information on the relevant variable was available

### Treatment modalities

Surgery was performed on 258/260 (99.2%) of the patients. Up to 187/258 (72.5%) of these patients underwent modified radical mastectomy. Chemotherapy was employed in 147/260 (56.5%) of the cohort and was adjuvant in 107 cases (72.8%). Of the 260 patients included, 129 (49.8%) effectively used hormone therapy. Amongst these, there were 13 patients (10%) who had completed cancer treatment and had used hormone therapy within a period of 5 years while the other 116 patients (90%) were still on hormone therapy during the period under study. Radiation therapy was delivered by a cobalt unit and was used in up to 201/260 (77.3%) in the postoperative period. The dose of 50Gy over a mean duration of 5 weeks ± 0.6 was given for most mastectomy patients when indicated. A dose of 50Gy over a 5 weeks period with a booster dose of 10Gy to the tumor site was administered if the histopathological analysis revealed positive surgical margins. The most frequent combination therapy was surgery, chemotherapy and radiation therapy which was employed in 66/260 cases (25.7%). This was closely followed by the combination of surgery, chemotherapy, radiation therapy and hormone therapy which was used in 62/260 cases (24.1%). Table 3 summarizes the treatment characteristics in our cohort.

**Table 3:** Treatment strategies used among patients with non-metastatic breast cancer

Variable	Category	Frequency (%)
Surgery	Yes	258(99.2)
	No	2(0.8)
		***260(100)***
Type of surgery	Radical mastectomy	187(72.5)
	Breast conserving surgery	71(27.5)
		***258(100)***
*Axillary lymph node dissection	Yes	210(81.4)
	No	48(18.6)
		***258(100)***
Chemotherapy	Yes	147(56.5)
	No	113(43.5)
		***260(100)***
Type	Neoadjuvant	14(9.5)
	Adjuvant	107(72.8)
	Both	26(17.7)
		***147(100)***
Drug combination	Cyclophosphamide + Doxorubicin + 5-Fluorouracil	120(82)
	Cyclophosphamide + Methotrexate + 5-Fluorouracil	7(4.8)
	Docetaxel + Doxorubicin + Cyclophosphamide	4(2.7)
	OTHERS	16(10.8)
		***147(100)***
**Hormone therapy**	Yes	129(49.8)
	No	131(50.4)
**Type**	Anastrozole	7(5.4)
	Tamoxifen	122(94.6)
		***129(100)***
Duration	5years	13(100)
Radiation therapy	Yes	201(77.3)
	No	59(22.7)
		***260(100)***
Duration	5 weeks	178(88.6)
	6 weeks	20(10.0)
	9 weeks	2(1.0)
	10 weeks	1(0.5)
		***201(100)***
Treatment combination		
Surgery + chemotherapy + radiation therapy		66(25.7)
Surgery + chemotherapy + radiation therapy + hormonal therapy		63(24.1)
Surgery + radiation therapy + hormonal therapy		50(19.5)
Surgery alone		32(12.5)
Surgery + radiation therapy		20(7.8)
Surgery + chemotherapy		12(4.7)
Surgery + hormonal therapy		11(3.9)
Surgery + chemotherapy + hormonal therapy		6(2.0)
		***260(100)***

The entries in boldface/italics represent the total case files for which information on the relevant variable was available

### Clinical progress of the study population

Table 4 describes the clinical progress of the study population. The median follow up period was 24 months (range: 3 months to 168 months). Overall, recurrences were noted in as many as 142/260 cases (54.6%). Amongst these, 68 (26.2%) developed local recurrences that were noted between 1 to 120 months (median recurrence time of 13 months) and 74 (28.5%) developed distant metastases that were noted between 1 month to 108 months (median time of 12 months) after initial treatment. During follow up, 24/260 patients (9.2%) developed bone metastasis followed by lung metastasis in 22/260 cases (8.5%), brain metastasis in 14/260 cases (5.4%) and liver metastasis in a small minority of 7/260 cases (2.7%). Nodal metastasis was noted in 18/260 cases (6.9%). A few cases (4.7%) developed metastasis to multiple organs. As concerns metastasis to bone, recurrences were observed between 1 to 60 months (median period of 12 months) after initial treatment. As concerns lung metastasis, the time between initial treatment and occurrence of metastasis ranged from 1 to 72 months with a median period of 20 months. The time elapse between initial treatment and the occurrence of brain metastasis ranged from 3 months to 36 months (median period of recurrence of 12 months). In relation to liver metastasis, its occurrence was noted between 2 to 108 months (median period of 40 months) after initial treatment. The time intervals between primary treatment and metastatic recurrences are summarized on Table 5.

**Table 4:** Clinical progress of study population

Adverse event	Frequency (%)
Local recurrence	68(26.2)
Distant metastases	74(28.5)
All cases of recurrence	142(54.6)
Total case files included	260(100)
Sites of recurrence	
Chest wall	68(26.2)
Brain	14(5.4)
Bone	24(9.2)
Liver	7(2.7)
Lung	22(8.5)
Lymph nodes	18(6.9)
Bone and brain	2(0.8)
Bone and lung	5(1.9)
Liver and lung	1(0.4)
Brain and lung	3(1.2)
Brain and liver	1(0.4)
Sequelae	
1. Lymphatic: lymphoedema of arm	17(6.5)
2. Pulmonary: lung fibrosis	4(1.5)
3. Neurologic: plexopathy	1(0.4)
4. Dermatologic: skin fibrosis	1(0.4)
radiodermatitis	1(0.4)
5. Vascular: telangiectasia	1(0.4)
6. Bone: rib fracture	1(0.4)
All cases with sequelae	26(10.8)

**Table 5:** Time interval between initial treatment and occurrence of metastases

Type of metastasis	Interval (months)	Frequency	Percentage
Local recurrence	0–12	38	55.9
	13–24	13	19.1
	25–36	4	5.9
	37–48	4	5.9
	49–60	3	4.4
	>60	6	8.8
		***68***	***100***
Distant metastasis	0–12	37	50
	13–24	17	23
	25–36	10	13.5
	37–48	4	5.4
	49–60	4	5.4
	>60	2	2.7
		***74***	***100***
Brain metastasis	0–12	7	50
	13–24	3	21.4
	25–36	4	28.6
		***14***	***100***
Bone metastasis	0–12	15	62.5
	13–24	6	25
	25–36	2	8.3
	37–48	0	0
	49–60	1	4.2
		***24***	***100***
Lung metastasis	0–12	9	40.9
	13–24	6	27.3
	25–36	3	13.6
	37–48	1	4.5
	49–60	2	9.1
	>60	1	4.5
		***22***	***100***
Liver metastasis	0–12	3	42.9
	13–24	1	14.3
	25–36	0	0
	37–48	0	0
	49–60	1	14.3
	>60	2	28.6
		***7***	***100***
Nodal metastasis	0–12	8	44.4
	13–24	4	22.2
	25–36	4	22.2
	37–48	2	11.1
		***18***	***100***

The entries in boldface/italics represent the total case files for which information on the relevant variable was available

Sequelae were recorded in 26/260 cases (10%). The time elapased between initial treatment and occurrence of sequelae ranged from 1 to 48 months (Table 6) with a mean period of 30 months. Lymphoedema was the most frequent late complication and occurred in 17/260 cases (6.5%). The swelling due to lymphoedema was noted after an average period of 16 months with the period of occurrence after treatment ranging from 1 to 36 months. Lung fibrosis was the second most common sequela and was noted in 4 cases (1.5%). It occurred on average 22 months after initial treatment.

**Table 6:** Time interval between initial treatment and occurrence of various sequelae

Variable	Period (months)	Frequency(Percentage of total)
Arm edema	<12	10(58.8)
	12–24	4(23.5)
	25–36	3(17.6)
		***17(100)***
Lung fibrosis	<6	2(50)
	6–12	1(25)
	>12	1(25)
		***4(100)***
Radiodermatitis	7	***1(100)***
Lung telangiectasia	24	***1(100)***
Nervous plexopathy	12	***1(100)***
Skin fibrosis	12	***1(100)***
Rib fracture	24	***1(100)***

The entries in boldface/italics represent the total case files for which information on the relevant variable was available

At the end of the period under review, 118/260 patients (45.4%) were alive and disease-free with a median follow up time of 24 months range (3 months to 168 months). We noted that 109/260 patients (41.9%) were followed-up for 12 months or less as shown on Table 7.

**Table 7:** Durations of follow-up among patients with non-metastatic breast cancer

Interval	Frequency	Percentage
0–12	109	41.9
13–24	64	24.6
25–36	26	10.0
37–48	18	6.9
49–60	17	6.5
>60	26	10.0
Total	260	100

A comparative table (Table 8) summarizes our main findings and compares these findings with those of some previous studies assessing the clinical progress of patients with NMBC within and outside Africa.

**Table 8:** Comparison of main findings of studies assessing the clinical progress of patients treated for non-metastatic breast cancer

Authors and location	Investigation	Study design and study population	Main findings
Millar et al. 2009, Australia [10]	Prediction of LR, DM, and Death After Breast-Conserving Therapy in Early-Stage Invasive Breast Cancer Using a 5-Biomarker Panel	Randomized clinical trial:498 cases	Median follow-up: 84 months. Ipsilateral breast tumour recurrence:24 (4.8%), LR:35 (7%), DM:47 (9.4%), and cancer deaths:37 (7.4%). Overall 5-year disease-free rates: ipsilateral breast tumour recurrence,97.4%; LR,95.6%; DM: 92.9%, and cancer–specific death:96.3%. Significant difference in survival between subtypes (of invasive breast cancer) for LR, DM and breast cancer–specific death
Diniz et al. 2016, Brazil (South America) [11]	Disease-free survival in patients with NMBC	Three-year retrospective single-centre study:563 cases	Disease recurrence noted in 129 cases:17.8% LR; 54.3% DM and 27.9% died. Disease free survival at 5 years:72%
Budakoglu et al. 2014, Turkey (Eurasia) [12]	Outcome of triple negative NMBC patients	Eleven-year multi-center retrospective study:561 cases	Ratio of triple-negative breast cancer:12%. Median patient follow-up was 28 months (range 3–290). Most commonly variant was invasive ductal carcinoma (84.1%). Grade II and III tumours were 27.1 and 48.5%, respectively. DM occurred in 134 (23.8%) patients and was mainly to bone, soft tissue, and lung. Factors affecting DFS and OS: age, lymph node involvement, lymphovascular invasion, tumour stage, adjuvant anthracycline-based chemotherapy and type of surgery (not significant for DFS). Three-year DFS and OS:72.0 and 93.0%, respectively
Jamshed et al. 2015, India (Asia) [13]	Clinical outcome of primary NMBC: A single institution experience	Fifteen-year single-center retrospective study:2829 cases	The median follow-up: 4.4 years. Recurrence following primary treatment was seen in 35% of the patients: 5% local, 1% regional, and 29% distant. At time of last follow-up: 960 patients were dead and 1869 (66%) were alive of which 112 were alive with disease. Cause of dead: breast cancer in 922 patients, treatment related toxicity in 8 patients and non-cancer related in 30 patients.
Alieldin et al. 2014, Egypt (North Africa) [14]	Age at diagnosis in women with NMBC: Is it related to prognosis?	One-year single-center retrospective study:941 cases	Most presented with advanced disease. All relapse: 44.2%; DM: 33.5%; LR: 6.6%. Women below 40 years of age had higher recurrence rates and poorer prognosis
Mathew et al. 2004, India (Asia) [15]	Do younger women with non-metastatic non-inflammatory breast carcinoma have poor prognosis?	Ten-year single-center prospective study:1701 cases	Median follow-up period: 66 months. Six hundred and forty (38%) were dead while 556 were not cured. Of the 556 patients, 125 (22.4%) had metastasis in bone (7.1% spine), 41 (7.3%) in liver, 40 (7.1%) in lung, 34 (6.1%) in brain, 20 (3.5%) in opposite breast and 169 (30.3%) had multiple metastasis. Women <40 years with T3/T4 breast lesions and/or positive axillary nodes had a significantly poorer survival
Our study, Cameroon (Central Africa)	Evolutionary aspects of NMBC after primary treatment in a sub-Saharan African setting	Sixteen-year single-center retrospective study:260 cases	Median follow up period: 24 months. Majority of patients had invasive ductal carcinoma. Metastases occurred in 142/260 (54.6%): 68/142 (26.2%) LR and 74/142 (28.5%) DM. DM: 9.2% bone, 8.5% lungs, 6.9% nodal, and 5.4% brain; 4.7% multiple metastasis. Median periods of occurrence of LR and DM: 3 and 12 months respectively. Sequelae were noted in 26/260 (10%), after an average of 30 months. Main sequelae: lymphedema (6.5%) and lung fibrosis (1.5%). At the end of review period, 118/260 patients (45.4%) were alive and disease-free.

*NMBC* Non-metastatic breast cancer, *LR* Local Relapse/Recurrence, *DM* Distant metastasis, *DS* Disease free survival, *OS* Overall survival

## Discussion

African countries are characterized by extreme paucity of reports on the morbidity of NMBC following various available treatment modalities [6]. This study is one of the rare assessing the outcome of NMBC in sub-Saharan Africa in an era of progressive health infrastructural development [7]. The study contributes to solving the problem of alarming lack of data and guidelines on cancer treatment in sub-Saharan Africa where the incidence of breast cancer is on the rise.

Our study revealed that patients with non-metastatic breast cancer were relatively young and this reflects the usual early age of onset of breast cancer among black Africans. Although the reason for this is poorly elucidated, it has been suggested that breast cancer seems to have a more aggressive propensity and consequently manifests earlier in Americans of black African descent [16]. Most patients in our study had invasive ductal carcinoma and this corroborates with the findings of previous reports from Africa [17–21]. Surgery remained the mainstay of treatment in our review and was typically non-conservative because of the prevailing locally-advanced breast cancer which may have been consequent to factors such as inadequate self-breast examination, poverty leading to delayed medical care, prolonged denial after diagnosis, fear of disfigurement from surgery and preferred alternative medicine [22]. The documented number of positive lymph nodes extracted during the surgical procedures was substantial and comparable to that of a previous report carried out in an urban setting like ours [20]. Albeit such extensive rates of positive lymph nodes could be accounted for by large tumour sizes at diagnosis as well as the aggressive variant and poor histological differentiation of breast cancer in most of the patients, it is worth noting that lymph node dissection and analysis in our setting was generally done for cases with clinical suspicion of advanced disease. This may have induced a bias towards overestimation of the pathological incidence of positive lymph nodes. Combinations of various treatment options were used in a greater majority of our cohort. Overall, after initial treatment, metastatic recurrences were frequent, even though most were local recurrences. Distant metastases usually affected bones and the lungs. Metastatic recurrences generally occurred few months after initial treatment contrary to the less observed sequelae that generally occurred much later and were mainly accounted for by lymphoedema and lung fibrosis.

The highest proportion of metastatic recurrences were observed within the first 24 months after primary treatment, which is fairly similar to the results obtained by *Noufel* et al. who observed the highest proportion of recurrences between 12 and 19 months [23]. Also, Donnelly et al. reported that median time to presentation of recurrence was 19 months for metastatic and 18 months for locoregional disease [24]. Chauleur et al. in France found that the period of observation for local recurrence ranged from 4 to 36 months, while distant metastatis ranged from 9 to 24 months [25]. While noting the relatively short median period of development of specific organ metastasis observed in our study, the findings in these reports and our review indicate the need to implement closer follow-up strategies especially during the first twenty four months after primary treatment since this is the period when recurrences tend to occur irrespective of the income setting. It was also noted that a considerable proportion of patients in our review presented for follow up only during the first 12 months after treatment. Aside poverty and ignorance of the seriousness of the disease, this phenomenon could be explained by the fact that after initial treatment, patients may underestimate the possibility of adverse events. This phenomenon further highlights the necessity to strengthen patient education, follow-up strategies and means of recapturing lost to follow up cases after primary treatment of NMBC.

Local recurrence was noted in 26.2% of our cases, a figure which is higher than those previously reported in low-income settings of Nigeria by Elumelu et al. (16%), Ethiopia by Ersumo et al. (15%), Afghanistan by Noufel et al. (7%), and India by Raina et al. (2.7%) [21, 23, 26, 27]. While the high rate of local recurrence in our study may be attributed to prevalent locally-advanced breast cancer and possibly non-adherence to adjuvant treatment which is known to significantly limit locoregional recurrence, it is worth noting that there were wide variations in sample sizes, clinico-pathological characteristics, treatment modalities and durations of follow-up in all the studies. These may account for the differences in the rates of local recurrences. The fair comparability in the rates of local recurrence in our report, that of Elumelu et al. in Nigeria and Ersumo et al. in Ethiopia, may be attributed to the similar ethnic and clinico-pathological characteristics as well as similar durations of follow-up of the study populations.

The proportion of patients with early breast cancer who develop distant metastases generally varies 15% to 25% at 5 years regardless of surgical technique [28]. In our report, the rate of distant metastasis was up to 28.5% which is relatively high in comparison to what was  reported in a previous study carried out in sub-Saharan Africa [29]. Apart from the high rate of locally-advanced tumour and the invasive nature of the histological type of breast cancer found in most of our patients, this observation could also be associated with the predilection of breast cancer for the upper outer quadrant in our study population. This quadrant is in close proximity to the axillary tail which is a more frequent channel for metastases when compared with the other quadrants of the breast [29, 30]. In spite of significant variations in reported rates, bones and lungs are generally the most frequent sites of distant metastasis in patients with breast cancer [31]. A previous study in Pennsylvania detected bone metastasis in up to 40% of a cohort of breast cancer patients [32] probably due to the longer follow-up period and better diagnostic resources.

The rate and mean period of occurrence of sequelae in our study is almost equivalent to what was reported by Hagigat et al. in Iran and Pawlaczyk et al. although they noted prevalence rates as high as 31.7% [33] and 22% [34] respectively. Furthermore, Kornblith et al. reported a prevalence rate of sequelae of up to 39% twenty years after the initial treatment [35]. Even though Hagigat et al. explained that postoperatively most patients usually limit movement of the surgical site which can induce lymphoedema [33] (the most frequent sequela in our study and previous reports), the relatively high rates of sequelae noted in these studies could be attributed to the longer follow-up periods and invariably lower rates of lost to follow-up which permitted detection of more sequelae.

The current report is not void of limitations. There was a possibility of selection bias given its retrospective single-center design. It is also worth mentioning that the stringent nature of our selection criteria resulted in a considerably low inclusion rate which was, nonetheless, necessary for reliable results to be generated from the poorly archived medical records in our context. Furthermore, our study like most from Africa, focused on describing morbidity data and lacked direct survival analysis. Nonetheless, we were able to derive that less than half of the patients were alive and disease-free at the end of the review period, albeit the individual follow-up periods were regrettably short in a significant proportion of the patients.

## Conclusions

Despite progressive health infrastructural development in sub-Saharan Africa, our study reveals that adverse events were frequent and occurred relatively early among patients who received primary treatment for NMBC during the 16 year period from 1997 to 2012 at the Douala General Hospital in Cameroon. The available cancer therapeutic modalities in this setting ought to be supplemented with guidelines on efficient strategies of follow-up and monitoring so as to optimize the care provided to these patients and ultimately improve on their survival. For these to be achieved, further studies on prognostic features of patients receiving primary treatment for NMBC in the sub-Saharan African context may be warranted.

## Abbreviation

NMBC: Non-metastatic breast cancer
